# Toward Plane‐Thickness‐Aliquot Matching in Dual‐Plane Biostimulator Injection

**DOI:** 10.1111/jocd.71068

**Published:** 2026-07-09

**Authors:** Jui‐Yu Lin, Chuan‐Yuan Lin

**Affiliations:** ^1^ Nanzi Annyeong Clinic Kaohsiung Taiwan; ^2^ Chengdu Royalty Aesthetic Clinic Chengdu Sichuan China; ^3^ Hangzhou Royalty Aesthetic Clinic Hangzhou Zhejiang China

**Keywords:** biostimulators, collagen stimulators, facial rejuvenation, injection technique, polycaprolactone, poly‐D,L‐lactic acid

To the Editor,

We read with great interest the article by Hong et al., entitled “A Dual‐Phase Approach to Facial Rejuvenation Integrating Contouring and Ligamentous (CL Code) Anchoring With a Polycaprolactone‐Based Collagen Stimulator,” published in the Journal of Cosmetic Dermatology [1]. The authors should be commended for presenting a structured and anatomy‐guided protocol that aligns contour restoration with deep support‐point treatment. The nomenclature is also elegant: “CL Code” concisely captures contouring and ligamentous anchoring while harmonizing with the polycaprolactone (PCL) material platform. This memorable framework may facilitate teaching, communication, and standardization in biostimulator‐based rejuvenation.

Structured anatomical coding systems have helped standardize injection location, depth, delivery technique, and volume in conventional volumizing filler treatment [2]. An important implication of the article by Hong et al. is that a similar coded and anatomy‐guided planning strategy can be extended to collagen‐stimulating fillers [1]. However, biostimulator protocols still lack standardized guidance on how injection plane, preparation thickness, and deposit size should be matched. Here, “thickness” is used as a practical descriptive term encompassing dilution‐ or concentration‐related changes in physical consistency, rather than as a single rheological parameter. We therefore propose the plane–thickness–aliquot relationship as a conceptual, hypothesis‐generating mechanistic model: superficial planes may favor thinner preparations with small aliquots for diffuse biostimulation, whereas deeper planes may favor thicker preparations with controlled focal aliquots for projection and support (Table 1). Regional anatomy may modify this relationship. Relevant factors include skin and soft‐tissue thickness, fat‐compartment compliance, vascularity, retaining‐ligament anatomy, and mobility of soft‐tissue compartments.

**TABLE 1:** Conceptual plane–thickness–aliquot relationship in dual‐plane biostimulator injection.

Injection plane	Tissue characteristics	Preferred suspension property	Aliquot strategy	Intended clinical role	Main safety consideration
Superficial plane (diffuse biostimulatory plane)	Subcutaneous or subdermal tissue; higher visibility of product irregularity	Thinner or lower‐concentration suspension; lower viscosity and cohesivity to facilitate broader and more even distribution	Small, widely distributed aliquots using fanning, threading, or evenly dispersed microdeposits	Diffuse collagen stimulation, skin quality improvement, soft‐tissue blending, and surface contour refinement	Avoid focal accumulation, papules, nodules, visible irregularity, discoloration, and uneven collagen stimulation
Deep plane (structural support plane)	Supraperiosteal or deep structural plane; greater relevance to projection, support, and contour restoration; tissue capacity varies by anatomical site	Thicker or higher‐concentration suspension; greater resistance to deformation, adjusted according to product formulation and treatment region	Controlled focal aliquots; deposit size should be limited and individualized according to injection depth, anatomical site, tissue capacity, and product rheology	Projection, contouring, structural support, and targeted collagen‐stimulating reinforcement	Avoid excessive bolus size, overcorrection, vascular‐risk zones, compression‐related effects, and product accumulation in confined spaces

*Note:* This table is intended as a conceptual framework rather than a validated dosing guideline. Specific dilution, injection plane, technique, and aliquot size should be individualized according to product formulation, anatomical region, tissue capacity, and physician experience.

This concept is consistent with experience using other biostimulators. A similar plane‐based strategy has been described in the AestheCode system for a poly‐D,L‐lactic acid (PDLLA)‐based biostimulator, in which deep structural injection uses thicker suspensions, whereas superficial wide‐zone biostimulation uses thinner suspensions [3]. A formulation‐specific example of this previously published PDLLA‐based approach is provided in Figure S1 and Table S1. Although PCL, PDLLA, poly‐L‐lactic acid, and calcium hydroxylapatite (CaHA) differ in formulation, degradation, carrier systems, handling characteristics, particle behavior, and host‐tissue responses, they share the need to match material behavior with tissue plane and treatment objective. Therefore, the proposed framework should not be transferred across products without formulation‐specific validation.

Mechanistically, thinner preparations may have lower viscosity or cohesivity, facilitating broader distribution in superficial planes. This may reduce focal accumulation and visible irregularity. Conversely, higher viscosity or cohesivity materials may better retain shape and provide structural projection in deeper planes. Recent CaHA rheological data support this principle, showing that dilution markedly lowers elastic modulus and shifts material behavior toward a more fluid‐like profile [4]. However, whether these rheological changes translate into differences in collagen stimulation, durability, or complication risk remains insufficiently validated. Thus, undiluted or minimally diluted preparations may be more suited to supraperiosteal contouring, whereas greater dilution may function mainly through biostimulation.

Deep‐plane injection also requires individualized aliquot control. Hong et al. appropriately used conservative deposits for deep‐plane support. Nevertheless, a universal deep bolus volume should be avoided. Temporal hollows, malar support points, and mandibular contouring sites differ in tissue thickness, compartment compliance, vascular proximity, and visibility of overcorrection. The optimal aliquot should therefore reflect injection depth, anatomical site, tissue capacity, desired projection, and product‐specific rheology. Needle/cannula position, injection speed, depth stability, and even distribution may also affect nodularity or uneven collagen deposition.

Safety considerations are product‐ and plane‐dependent. Because biostimulators exert delayed biological effects, potential adverse events may also include late‐onset nodules, inflammatory or granulomatous reactions, asymmetry, and unpredictable collagen remodeling. Persistent bruising or Tyndall‐like discoloration has recently been reported after liquid‐form PCL injection, particularly in thin or vascular regions. It was hypothesized to relate to pigment entrapment within a dense scaffold or superficial light‐scattering effects [5]. These findings highlight the importance of depth control, even distribution, cautious volume, and product‐specific risk assessment.

Another related consideration is the interpretation of the term “ligamentous anchoring,” which is clinically appealing and provides a useful anatomical framework for deep support‐point injection. At the present stage, this term may be best understood primarily as an anatomical and procedural descriptor, while future ultrasound, three‐dimensional volumetric analysis, or biomechanical studies may help determine whether targeted injection near ligamentous origins produces measurable reinforcement of retaining structures [6].

Overall, the CL Code is a valuable contribution to structured, anatomy‐guided biostimulator injection. The proposed plane–thickness–aliquot relationship is not a validated dosing guideline, but a preliminary framework for individualized treatment planning. Current limitations include the absence of controlled comparative trials, imaging confirmation of product distribution, quantitative plane‐specific collagen‐response data, and evidence of cross‐product transferability. Future studies should incorporate ultrasound, three‐dimensional volumetric analysis, histology when feasible, prospective comparisons of thickness strategies, and standardized adverse‐event reporting. Pending validation, and with individualized assessment remaining essential, it may help clinicians consider injection plane, material behavior, aliquot size, and safety risk together while future evidence determines whether plane‐based planning improves efficacy, safety, or reproducibility.

## Funding

The authors have nothing to report.

## Ethics Statement

The authors have nothing to report.

## Conflicts of Interest

Dr. Chuan‐Yuan Lin and Dr. Jui‐Yu Lin previously served as medical directors of REGEN Biotech. They declare no other conflicts of interest related to this work.

## Supporting information


**Figure S1:** Previously published PDLLA‐based dual‐plane treatment map. This Supplementary Figure reproduces a previously published AestheCode implementation for a PDLLA‐based biostimulator, including deep structural support points and superficial wide‐zone biostimulatory areas. It is provided only as a product‐specific example of plane‐specific treatment planning and should not be interpreted as a universal treatment map or dosing guide for PCL, PLLA, CaHA, or other biostimulatory fillers. CaHA, calcium hydroxylapatite; PCL, polycaprolactone; PDLLA, poly‐D,L‐lactic acid; PLLA, poly‐L‐lactic acid (Reproduced from Lin JY, Lin CY. *The AestheCode system: a safe and efficient guide for AestheFill injection*. Aesthetic Plast Surg. 2025;49:2658–2660. doi:10.1007/s00266‐024‐04250‐4, with permission).

**Table S1:** Previously published PDLLA‐based example of plane‐specific suspension thickness, injection layer, and aliquot strategy. This Supplementary Table is provided only as an example of a previously published PDLLA‐based dual‐plane implementation. The listed concentrations, planes, and aliquot sizes are product‐specific and should not be generalized to other biostimulators without formulation‐specific validation. PDLLA, poly‐D,L‐lactic acid (Reproduced from Lin JY, Lin CY. *The AestheCode system: a safe and efficient guide for AestheFill injection*. Aesthetic Plast Surg. 2025;49:2658–2660. doi:10.1007/s00266‐024‐04250‐4, with permission).

## Data Availability

Data sharing not applicable to this article as no datasets were generated or analysed during the current study.
