# Supplementary figures and images for: Toward Plane‐Thickness‐Aliquot Matching in Dual‐Plane Biostimulator Injection
Source: J Cosmet Dermatol. 2026 Jul 9;25(7):e71068. doi: 10.1111/jocd.71068 (PMC13351818; doi:10.1111/jocd.71068)

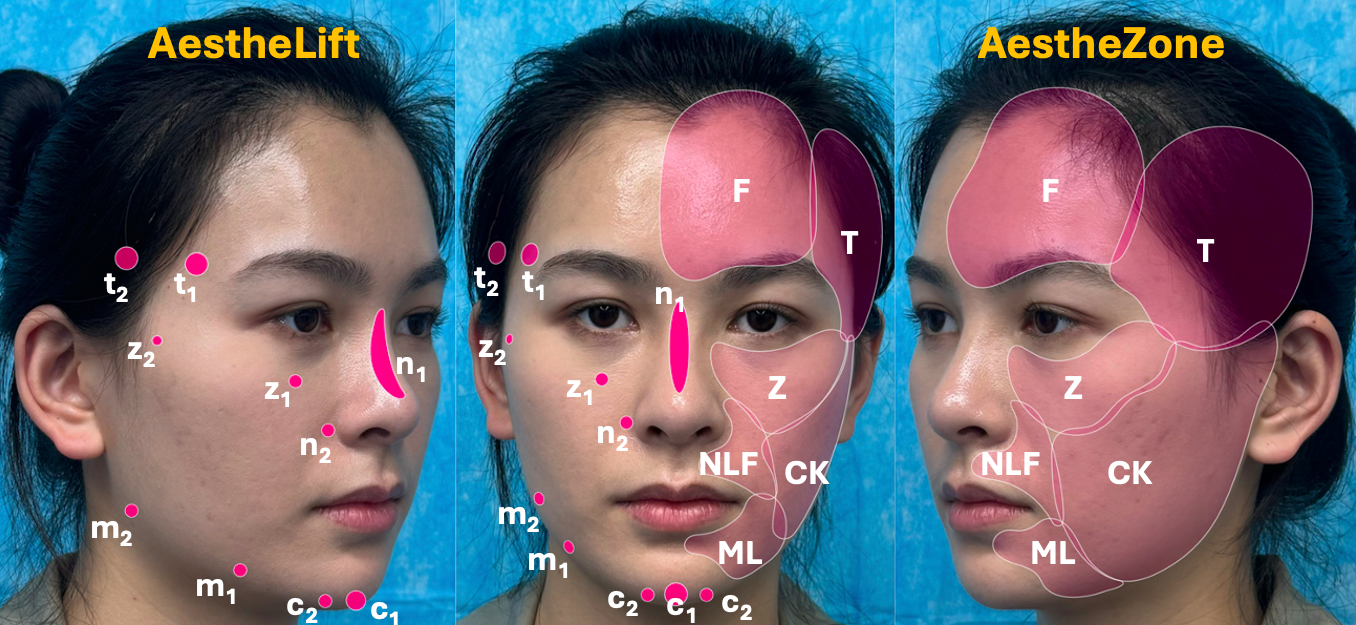

Supplement: Supplementary file 1 — Figure S1: Previously published PDLLA‐based dual‐plane treatment map. This Supplementary Figure reproduces a previously published AestheCode implementation for a PDLLA‐based biostimulator, including deep structural support points and superficial wide‐zone biostimulatory areas. It is provided only as a product‐specific example of plane‐specific treatment planning and should not be interpreted as a universal treatment map or dosing guide for PCL, PLLA, CaHA, or other biostimulatory fillers. CaHA, calcium hydroxylapatite; PCL, polycaprolactone; PDLLA, poly‐D,L‐lactic acid; PLLA, poly‐L‐lactic acid (Reproduced from Lin JY, Lin CY. The AestheCode system: a safe and efficient guide for AestheFill injection. Aesthetic Plast Surg. 2025;49:2658–2660. doi:10.1007/s00266‐024‐04250‐4, with permission). [file JOCD-25-e71068-s001.png]
