# Supplementary material for: Toward Plane‐Thickness‐Aliquot Matching in Dual‐Plane Biostimulator Injection
Source: J Cosmet Dermatol. 2026 Jul 9;25(7):e71068. doi: 10.1111/jocd.71068 (PMC13351818; doi:10.1111/jocd.71068)
Supplement: Supplementary file 2 — Table S1: Previously published PDLLA‐based example of plane‐specific suspension thickness, injection layer, and aliquot strategy. This Supplementary Table is provided only as an example of a previously published PDLLA‐based dual‐plane implementation. The listed concentrations, planes, and aliquot sizes are product‐specific and should not be generalized to other biostimulators without formulation‐specific validation. PDLLA, poly‐D,L‐lactic acid (Reproduced from Lin JY, Lin CY. The AestheCode system: a safe and efficient guide for AestheFill injection. Aesthetic Plast Surg. 2025;49:2658–2660. doi:10.1007/s00266‐024‐04250‐4, with permission). [file JOCD-25-e71068-s002.pptx]

## Slide 1
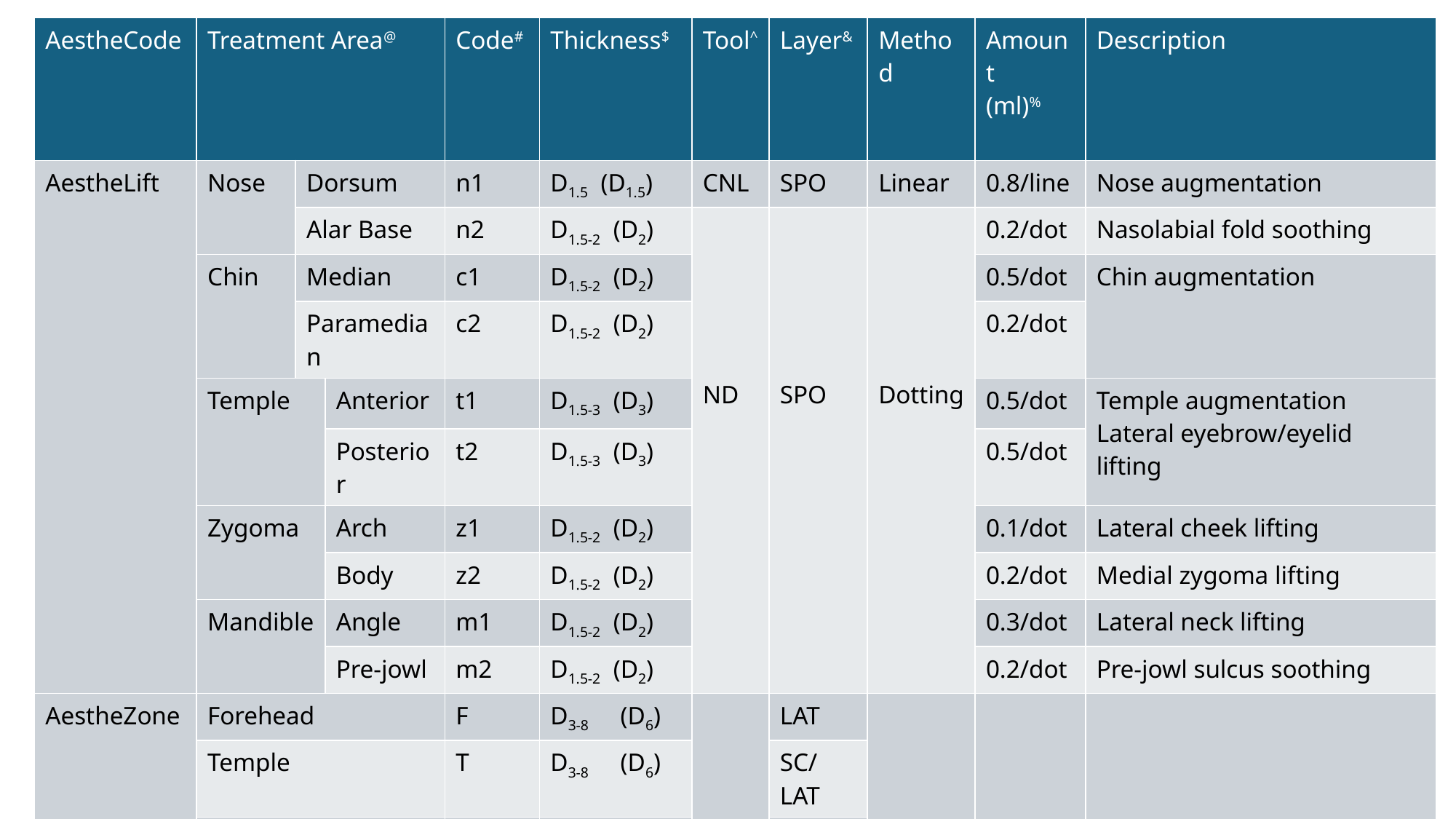

| AestheCode | Treatment Area@ | | | Code# | Thickness$ | Tool^ | Layer& | Method | Amount (ml)% | Description |
| --- | --- | --- | --- | --- | --- | --- | --- | --- | --- | --- |
| AestheLift | Nose | Dorsum | | n1 | D1.5 (D1.5) | CNL | SPO | Linear | 0.8/line | Nose augmentation |
| | | Alar Base | | n2 | D1.5-2 (D2) | ND | SPO | Dotting | 0.2/dot | Nasolabial fold soothing |
| | Chin | Median | | c1 | D1.5-2 (D2) | | | | 0.5/dot | Chin augmentation |
| | | Paramedian | | c2 | D1.5-2 (D2) | | | | 0.2/dot | |
| | Temple | | Anterior | t1 | D1.5-3 (D3) | | | | 0.5/dot | Temple augmentation Lateral eyebrow/eyelid lifting |
| | | | Posterior | t2 | D1.5-3 (D3) | | | | 0.5/dot | |
| | Zygoma | | Arch | z1 | D1.5-2 (D2) | | | | 0.1/dot | Lateral cheek lifting |
| | | | Body | z2 | D1.5-2 (D2) | | | | 0.2/dot | Medial zygoma lifting |
| | Mandible | | Angle | m1 | D1.5-2 (D2) | | | | 0.3/dot | Lateral neck lifting |
| | | | Pre-jowl | m2 | D1.5-2 (D2) | | | | 0.2/dot | Pre-jowl sulcus soothing |
| AestheZone | Forehead | | | F | D3-8 (D6) | CNL | LAT | Fanning | 0.1/cm2 | Wrinkles/Depressions soothing Fine lines/Pores improvement |
| | Temple | | | T | D3-8 (D6) | | SC/LAT | | | |
| | Zygoma | | | Z | D4-12 (D8) | | SC/SD | | | |
| | Cheek | | | CK | D4-12 (D8) | | SC | | | |
| | Naso-labial Fold | | | NLF | D4-12 (D8) | | SC | | | |
| | Marionette Line | | | ML | D4-12 (D8) | | SC | | | |

## Slide 2
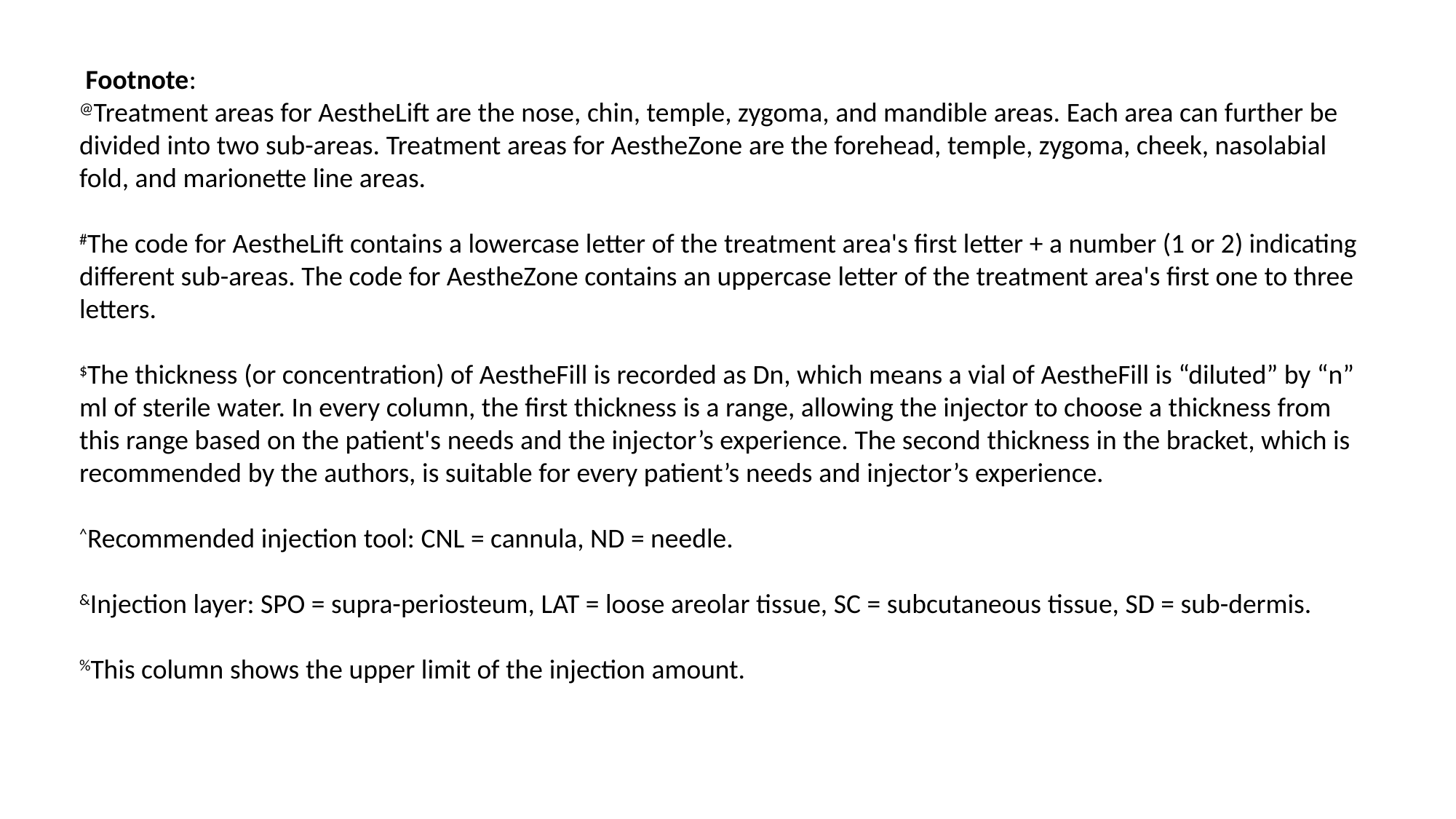

Footnote:
@Treatment areas for AestheLift are the nose, chin, temple, zygoma, and mandible areas. Each area can further be divided into two sub-areas. Treatment areas for AestheZone are the forehead, temple, zygoma, cheek, nasolabial fold, and marionette line areas.
#The code for AestheLift contains a lowercase letter of the treatment area's first letter + a number (1 or 2) indicating different sub-areas. The code for AestheZone contains an uppercase letter of the treatment area's first one to three letters.
$The thickness (or concentration) of AestheFill is recorded as Dn, which means a vial of AestheFill is “diluted” by “n” ml of sterile water. In every column, the first thickness is a range, allowing the injector to choose a thickness from this range based on the patient's needs and the injector’s experience. The second thickness in the bracket, which is recommended by the authors, is suitable for every patient’s needs and injector’s experience.
^Recommended injection tool: CNL = cannula, ND = needle.
&Injection layer: SPO = supra-periosteum, LAT = loose areolar tissue, SC = subcutaneous tissue, SD = sub-dermis.
%This column shows the upper limit of the injection amount.
